# Driving down the Detection Limit in Microstructured Fiber-Based Chemical Dip Sensors

**DOI:** 10.3390/s110302961

**Published:** 2011-03-04

**Authors:** Erik P. Schartner, Heike Ebendorff-Heidepriem, Stephen C. Warren-Smith, Richard T. White, Tanya M. Monro

**Affiliations:** Institute for Photonics & Advanced Sensing, School of Chemistry & Physics, University of Adelaide, Adelaide, SA 5005, Australia; E-Mails: heike.ebendorff@adelaide.edu.au (H.E.-H.); stephen.warrensmith@adelaide.edu.au (S.C.W.-S.); richard.white@adelaide.edu.au (R.T.W.); tanya.monro@adelaide.edu.au (T.M.M.)

**Keywords:** soft glass, dip sensing, microstructured optical fiber, quantum dots

## Abstract

We present improvements to fluorescence sensing in soft-glass microstructured optical fibers that result in significantly improved sensitivity relative to previously published results. Concentrations of CdSe quantum dots down to 10 pM levels have been demonstrated. We show that the primary limitation to the sensitivity of these systems is the intrinsic fluorescence of the glass itself.

## 1. Introduction

Optical fibers are ideal for environmental sensing applications because of their ability to transmit optical signals to and from the sensing region without the use of free-space optics. By accessing the evanescent field, the fiber itself can be the sensing element and long interaction lengths can be achieved [1]. Microstructured optical fibers (MOFs) are particularly suited to such applications as the air spaces inside the fiber form natural cavities for locating the material to be detected. These types of fibers have a significant advantage over conventional core-clad fibers, in that they can be fabricated from a single material, so issues involving thermal and chemical compatibility between different glasses can be avoided [2].

By tailoring both the MOF material and the geometry, the light-matter overlap can be increased to values much larger than with conventional fibers. Through varied structure geometries such as photonic band-gap fibers (PBGF) [3,4] or suspended nanowires [5–8], the overlap between the guided light and the analyte located within the holes of the fiber can be increased significantly over that which can be obtained using multi-mode bare fibers or D-shaped fibers [9]. However, due to the relatively limited bandwidth of most PBGFs [7], the excitation and emission wavelengths must be relatively close to enable detection with the fiber. Here we employ the suspended nanowire design [10] that provides the high evanescent overlap of a standard nanowire [11] with the large interaction length and robust handling comparable to conventional fibers.

The extremely small transverse structures that are possible in MOFs allow very small samples to be measured, with total volumes of less than 10 nL being easily obtainable with practical (∼20 cm) lengths of fiber using the fiber shown in Section 3 below.

Through the use of fluorescent molecules that interact with target species these fibers can be applied to diverse applications including hydrogen peroxide detection [12] or aluminum detection [10]. The detection of biomolecules attached to fluorescent labels has recently been demonstrated in suspended-core MOFs [3,13], yielding a detection sensitivity down to 1 nM for antibodies labelled with quantum dots (Qdots).

The fluorescence-detection approach is attractive because of its simplicity. When one end of the fiber is dipped into the sample, capillary forces draw the liquid into the voids within the fiber. The evanescent field of the pump light excites the fluorescent labels and a portion of the fluorescence is captured by the fiber core and propagates to the fiber tips. Captured fluorescence can be detected at either end of the fiber, although backward detection provides the convenience of single-ended devices and an improved signal-to-pump ratio [14].

This can be done either for labelled biomolecules in solution [3,13], or, if specificity is required, by attaching recognition antibodies to the internal fiber surface [15,16]. In the later case, fluorescence is detected when antigens bind to their corresponding immobilized antibodies and non-bound antigens are flushed out of the fiber. In either case, efficient fluorescence-based MOF sensors require a large evanescent field in the fiber holes, such as in band-gap fibers, liquid-core fibers or suspended core fibers.

Suspended-core fibers are clearly a powerful platform both for chemical and biological sensing [10]. The aim of this work is determine the factors currently restricting the detection limit in this type of sensor and to improve this sensing architecture to increase the sensitivity of the dip sensor.

## 2. Glass Choices for Fluorescent Sensing Architectures

Various glasses were examined to test their suitability as a material for fabricating sensing fibers. The primary consideration here was the amount of fluorescence that was generated within the glass itself when light at the fluorophore’s absorption wavelength is guided within the fiber, as this has been identified as the primary limitation to the sensitivity of this type of sensor. The glass choice was restricted to soft glasses that can currently be fabricated into optical fibers through extrusion at temperatures less than 650 °C [2], so that suspended-core fibers could be fabricated via use of extruded preforms.

**Figure 1.**
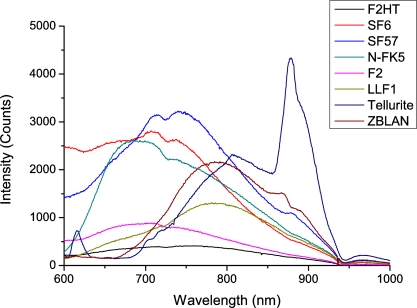
Bulk glass fluorescence measurements with 532 nm excitation.

To measure the glass background fluorescence spectra, a 25 mW 532 nm laser source was used to illuminate a range of bulk glass samples, and the fluorescence captured using a multi-mode fiber and recorded using an iHR 320 monochromator with the pump blocked with a long-pass filter. The results for various soft-glasses are shown in Figure 1 below. This fluorescence is believed to originate from trace metal impurities within the glass, although the exact origin of this fluorescence has not been studied in detail.

Of greatest interest to this work is the glass fluorescence background in the region of the Qdot 800 emission (as described below), which is between 700 and 900 nm, with a peak at 780 nm. These results suggest that the best two glasses are both commercial lead-silicate glasses from Schott; the F2 glass used in earlier [17] work, and the F2HT glass proposed for use here. A third candidate in LLF1, another Schott lead silicate which has also found extensive use in fabricating soft glass fibers and is considered in the modeling results shown in Section 7.

The bulk measurements suggest that since all other relevant material parameters are virtually identical between the two different glasses, changing glass from F2 to F2HT should result in almost a 50% reduction in the observed glass fluorescence.

## 3. Experimental Design

A commercially available CdSe quantum dot Qdot® 800 ITK^TM^ from Invitrogen was selected for use in this research. Unlike conventional organic fluorophores, quantum dots are virtually immune to the effects of photobleaching, meaning that direct comparisons between samples can be readily made as the fluorescence signal does not decrease over time. Additionally, due to the optical characteristics of Qdots, it is possible to excite the molecules at a wavelength that is significantly shorter than their fluorescence emission wavelength (Δ∼250 nm separation in this case: excitation at 532 nm, emission peak at 780 nm), which reduces the need to spectrally filter out residual pump light. Finally, they have a relatively high absorption coefficient (∼4 × 10^6^ M^−1^ cm^−1^ at 532 nm) compared to other organic flurophores such as Rhodamine B which has an absorption coefficient of 8.2 × 10^4^ at 532 nm. The Qdots in this case were unconjugated, and suspended in decane (CH_3_-(CH_2_)_8_-CH_3_). Note that conjugated Qdots could readily be used, and indeed protein detection down to 1 nM has previously been demonstrated in fiber using a similar system to that described here [13].

**Figure 2.**
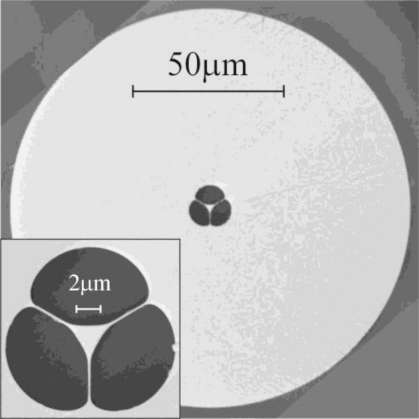
Scanning Electron Microscope (SEM) image of the soft glass MOF used for these experiments. The central, structured region of the fiber was made from F2HT glass, and the cladding from F2 glass.

The fiber used for these experiments was an in-house fabricated suspended core MOF as shown in Figure 2, referred to hereafter as a wagon-wheel (WW) fiber. The fiber was fabricated via preform extrusion and subsequent fiber drawing performed using a cane-in-tube technique [17] to allow the production of a relatively small core within a robust fiber geometry (core diameter ∼1.7 μm, outer diameter 130 μm). To the best of our knowledge this is the first MOF reported to have been fabricated from Schott F2HT glass [18], with the primary difference between this glass and the more commonly used F2 [14,19,20] being improved UV transmission [18] and a reduced intrinsic glass fluorescence, which was discussed in detail in Section 2. F2HT glass was used as the material for the central cane, with the outer tube being fabricated from standard F2 glass. As the thermal and chemical properties of the two glasses are virtually identical, no issues were seen to arise from this. The fabricated fiber had a loss of 0.9 dB/m at 532 nm.

## 4. System Characterization

**Figure 3.**
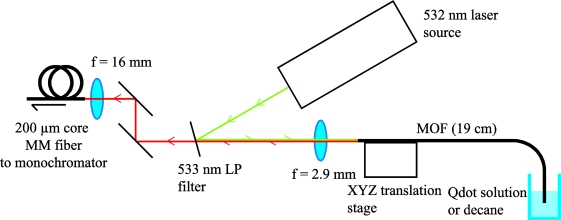
Experimental configuration for fiber-based fluorescence measurements.

Initial characterization was performed on the MOF using two different samples: a 1 nM Qdot 800 solution in decane, and plain decane without added Qdots. The fibers were filled simply by dipping the tip in the solution and allowing capillary forces to draw the liquid within the holes along the fiber length. Before measurements were taken the fiber was removed from the solution, and its alignment optimized by monitoring the laser power from the distal end of the fiber using a power meter as shown in Figure 3. Due to the small size of the holes within the fiber (∼4 μm) compared to the filled length (∼ 19 cm) it was observed that the effects from evaporation of the solvent were negligible as minimal amounts were lost even over a period of several days.

**Figure 4.**
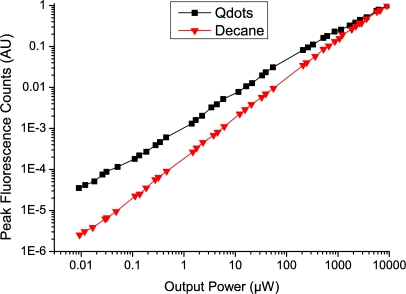
Peak fluorescence counts at 780 nm of both a fiber filled with a solution of Qdots in Decane (black squares) and a F2HT-core WW fiber filled with plain decane (red triangles) without Qdots added. The fluorescence counts have been normalized to 1 at 8.95 mW output power.

Results (as shown in Figure 4) were taken using a 25 mW 532 nm laser source, an iHR 320 monochromator and 533 nm long pass filter to remove any scattered or reflected pump light from the system. The filter was used as close to normal incidence as possible, and even when misaligned still demonstrated a greater than OD4 reduction for the pump light. The fiber was aligned by optimizing the output power. Minimizing the path length of the laser ensured that the alignment stability of the light coupled into the fiber was good, with a maximum power reduction of 10% being recorded over a period of one hour. Here we define the output power as the total pump power recorded from the distal end of the fiber. For reference the total coupling efficiency from the laser source to the core of the WW fiber is in the range 35–40%.

The aim of these experiments was to investigate and characterize the power dependence of both the background glass fluorescence and Qdot fluorescence to determine the optimal excitation power for the sensor.

The results in Figure 4 show that while the background glass fluorescence, as shown by the red symbols in Figure 4, increases almost linearly with the output power of the fiber, as might be expected, the Qdot signal is not linear with the output power. Note the logarithmic scale of the axes in Figure 4. As can be seen from Figure 4, as the power within the fiber increases, the Qdot signal decreases relative to the background glass fluorescence. A similar effect is observed using laser excitation on a 1 nM Qdot sample in cuvette giving a result that agrees within the error margin of the experiments with the result shown above. As such, when the power launched in to the fiber increases, the net sensitivity of the sensor decreases.

Previously published results [13] employed an optical spectrum analyzer, whereas this paper uses a monochromator/CCD setup, resulting in a significant improvement in the signal to noise ratio. As a result, it is now possible to perform measurements with less than 30 nW of input power (*i.e*., less than 10 nW output), whereas previous results required several milliwatts of input power to record any significant signal. This gives an immediate improvement to the Qdot fluorescence: glass fluorescence ratio of at least 5× which likewise corresponds to a 5× increase in the sensitivity of the system. Section 5 explores the improvements derived from these results.

## 5. Sensing Results

**Figure 5.**
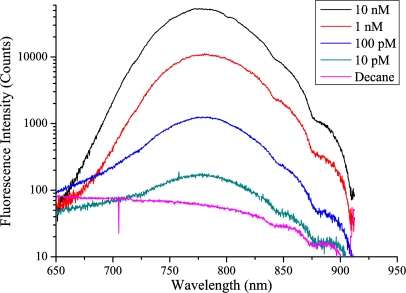
In-fiber fluorescence measurements with varied Qdot concentrations. As per previous results, a fiber filled with a plain decane solution was used to give a background reference signal (in this case the fluorescence comes from the glass itself).

A spectrum was taken of the unfilled fiber to ensure consistency between results to eliminate any possible variations in the signal arising from cleave-related reductions in the coupling efficiency, and the fiber filled with Qdots (or decane for the background signal) for a range of different concentrations. The results of these measurements are shown in Figure 5.

These results demonstrate that with a relatively high (compared to that shown in Figure 4) input power of 2.5 μW for all measurements, the minimum detectable Qdot signal using this configuration is 10 pM, which is a significant improvement over the 1 nM concentration previously reported in [13]. Given that the total volume of the fluid within the fiber used in this experiment is approximately 9 nL for the 19 cm fiber length, this implies that for the 10 pM minimum concentration demonstrated here there are in the order of 5 × 10^4^ Qdots located within the fiber.

## 6. Theoretical Modelling

Previous work [13] demonstrated that the fundamental limitation to the sensitivity of fiber-based fluorescence sensors is the background fluorescence from the glass itself, which was discussed in detail in Section 2. To better understand the behaviour of these sensors, we chose to model the system using the analytic vectorial solutions of a simple step-index fiber, as opposed to a FEM model [21], making the reasonable assumption that the supporting struts within the WW fiber have a negligible impact on the field distributions and fluorescence recapture. We define the effective core size as the diameter of the circle with an area equivalent to the largest triangle that fits wholly within the core [17]. For the purposes of this modeling, the fiber loss was ignored, as since both the glass fluorescence and Qdot signal fluorescence are located in the same spectral region, they both experience a comparable reduction in signal under transmission. As such, the primary effect of loss on this system is a reduction in the measured signal to noise ratio.

We seek to gain an understanding of how the impact of the undesirable glass fluorescence on the sensor performance can be minimized. To do this, we define a figure of merit (FOM) for both the background glass fluorescence (FOM_glass_) and for the Qdot signal fluorescence (FOM_signal_) as a measure of the intensity of the fluorescence that is captured into the backward propagating modes of the fiber. These FOMs give a measure of the total amount of fluorescence photons that are initially emitted and then recaptured in to the modes of the fiber. The former is a result of the amount of incident laser power on the fluorophores, and the latter due to the efficiency that photons emitted by the fluorophore are recaptured.

For the Qdot fluorescence, FOM_signal_ is defined as the product of the power fraction (PF) of the fundamental mode at the excitation wavelength located within the holes of the fiber (PF_clad_) [19] and the fluorescence capture fraction (FCF) of the emitted fluorescent light back in to all guided modes of the fiber [19].

Only the fundamental mode was considered for the excitation of the fluorescence, as through proper lens choice and careful alignment the laser light can be preferentially coupled into the fundamental mode of the fiber, even though the waveguide is capable of supporting higher order modes (HOMs) unless impractically small core sizes are considered.

**Figure 6.**
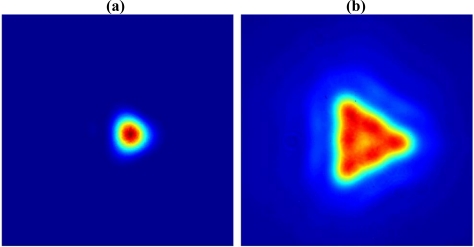
**(a)** Mode image of 532 nm excitation. Fiber filled with decane to ensure correct mode profile for comparison to fluorescence mode image. **(b)** Mode image of very high concentration (1 μM) Qdot 800 fluorescence mode excited with 532 nm source. Scales are directly comparable between (a) and (b).

For the fluorescence capture, it has been demonstrated that higher order modes must be included in the model, as the fluorescence capture into these modes has a significant impact on the total FCF, especially once the core size is increased well beyond the cut-off for single mode guidance [20]. Further supporting this, the experimentally measured mode images (Figure 6 below) show that while the excitation at 532 nm [Figure 6(a)] is well confined to the core and displays no obvious HOM content, the fluorescence emission around 780 nm [Figure 6(b)] clearly has substantial HOM content.

Similarly for the glass fluorescence, the same definitions and assumptions are made, with FOM_signal_ being defined as the product of the PF within the glass (*i.e.*, 1 − PF_clad_) and the FCF of the glass emitted light captured by all backward travelling modes of the waveguide.

**Figure 7.**
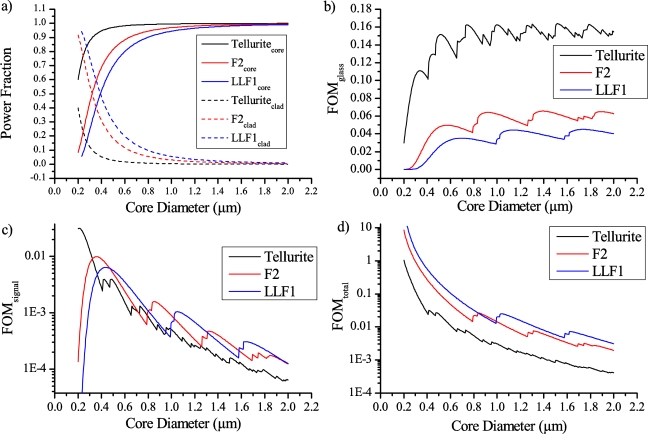
**(a)** Power fraction at the excitation wavelength located within the cladding or core for various glasses, decane filled. **(b)** Figure of Merit (FOM) for fluorescent photons excited within the core of the fiber, this represents the intrinsic glass fluorescence. **(c)** FOM for fluorescent photons excited within the holes—this represents the Qdot fluorescence. **(d)** Total FOM for the sensing system, with a higher value corresponding to a higher Qdot signal fluorescence relative to the background glass fluorescence.

These quantities can now be brought together to provide a better understanding of the overall performance of the sensor. This is done by defining a figure of merit, FOM_total_ = FOM_signal_/FOM_glass_. The primary constraint currently limiting the minimum detectable concentration of the sensor is the amount of Qdot signal fluorescence relative to the glass fluorescence. FOM_total_, shown in Figure 7(d), is a direct measure of the relative magnitude of detected Qdot signal relative to the glass that is observed by the sensor.

As can be seen from Figure 7, reducing the core size fiber has a significant impact on FOM_total_ (regardless of the glass choice), and thus on the performance of the sensor, with improving performance predicted for the smallest core sizes. However, it is important to note that loss mechanisms were not included in this study. It has been previously shown [17] that the loss increases significantly due to scattering from surface roughness both in tapered nanowires and suspended nanowires once the core size becomes sufficiently small. Additionally, modes close to cutoff also experience significant confinement loss [20], resulting in both a reduction in signal at small core sizes where FOM_total_ is predicted to be largest using the simple model derived here, and a reduction in signal at each point where an additional mode is guided as modes close to cutoff are generally spread out and not well confined.

The final parameter examined within this theoretical study was the effect of the index contrast between the glass and the cladding (Figure 7). Three glasses were examined: F2, LLF1 and Tellurite. The first two are commercially available lead silicates, with the third an in-house fabricated glass, with refractive indices at 532 nm of 1.63, 1.55 and 2.02 respectively. The refractive index used for the decane within the holes was 1.41, giving Δn values of 0.22, 0.14 and 0.61 respectively.

As shown from the plots in Figure 7 reducing the index contrast results in an increase in FOM_total_ which corresponds to an increase in the amount of Qdot signal fluorescence observed relative to the background glass levels. However when the intensity of the glass fluorescence for each of the three glasses (as shown in Figure 1) is considered the F2 or F2HT glasses will in practice show the lowest background glass signals and therefore the best sensitivity. This is a consequence of the F2HT fluorescence intensity being 3.3× lower than LLF1 at 780 nm, and 4.6× lower than the Tellurite fluorescence at the same wavelength. So even though the overall capture of the fluorescence photons is reduced for the LLF1 glass, since the efficiency with which they are generated within the glass is significantly higher the net result in fiber is a higher glass fluorescence signal. This is further exacerbated by using a higher index glass such as Tellurite, as more fluorescence is generated in the first place and since FOM_total_ is significantly lower with the higher index glass this results in a very large glass fluorescence signal relative to the Qdot signal.

These results demonstrate that a reduction in the index contrast results in a decrease in FOM_glass_, and depending on the core size can also show an increase in FOM_signal_ leading to an overall increase in FOM_total_.

## 7. Conclusion

We have demonstrated significant improvements in the sensitivity of fiber based fluorescence sensors, resulting in a large decrease in the minimum detectable concentration to 10 pM from the previously published result of 1 nM. This has been enabled through improvements to the experimental configuration and the use of low-fluorescence glass for the fiber fabrication, although we have demonstrated theoretically that this could be improved further by modifications to the fiber structure.

Although improvements in FOM_total_ can be obtained by reducing the core size, this also has the effect of making the experiment highly dependent on the coupling alignment, making reproducible results more difficult to come by as well as increased loss arising from surface roughness as mentioned earlier. Additionally, since the next phase of this work will involve splicing to conventional Silica SMF for easier integration and field deployability using extremely small core (*i.e.*, single mode at the fluorescence wavelength) results in a significantly reduced coupling efficiency between the silica fiber and the WW fiber.

By building on the improvements discussed within this paper, it should be possible to further reduce the lowest detectable concentration using MOF sensors of this type significantly beyond the results that have been demonstrated here. If a relatively low loss fiber with a smaller core size than used above were fabricated, the amount of observed glass fluorescence would drop significantly, enabling a further reduction in the detectable concentration to be observed. At present with a 10 pM concentration approximately 5 × 10^4^ Qdots are filled within the holes, and we anticipate that reducing the core size well below a micron would enable a drop in concentration to at least 1 pM with as few as 500 total Qdots within the fiber.
